# Spatiotemporal Divergence of the Warming Hiatus over Land Based on Different Definitions of Mean Temperature

**DOI:** 10.1038/srep31789

**Published:** 2016-08-17

**Authors:** Chunlüe Zhou, Kaicun Wang

**Affiliations:** 1 College of Global Change and Earth System Science, Beijing Normal University, Beijing, 100875, China; 2 Joint Center for Global Change Studies, Beijing 100875, China

## Abstract

Existing studies of the recent warming hiatus over land are primarily based on the average of daily minimum and maximum temperatures (*T*_*2*_). This study compared regional warming rates of mean temperature based on *T*_*2*_ and *T*_*24*_ calculated from hourly observations available from 1998 to 2013. Both *T*_*2*_ and *T*_*24*_ show that the warming hiatus over land is apparent in the mid-latitudes of North America and Eurasia, especially in cold seasons, which is closely associated with the negative North Atlantic Oscillation (NAO) and Arctic Oscillation (AO) and cold air propagation by the Arctic-original northerly wind anomaly into mid-latitudes. However, the warming rates of *T*_*2*_ and *T*_*24*_ are significantly different at regional and seasonal scales because *T*_*2*_ only samples air temperature twice daily and cannot accurately reflect land-atmosphere and incoming radiation variations in the temperature diurnal cycle. The trend has a standard deviation of 0.43 °C/decade for *T*_*2*_ and 0.41 °C/decade for *T*_*24*_, and 0.38 °C/decade for their trend difference in 5° × 5° grids. The use of *T*_*2*_ amplifies the regional contrasts of the warming rate, i.e., the trend underestimation in the US and overestimation at high latitudes by *T*_*2*_.

Land surface air temperature (*T*_*a*_) is one of the fundamental variables in weather and climatic observations, modeling, and applications^1^,^2^. Despite the ongoing increase in atmospheric greenhouse gases, the global mean surface temperature (GMST) has remained rather steady and has even decreased in the central and eastern Pacific since 1998^3^. This cooling trend is referred to as the global “warming hiatus”^4^,^5^. Several explanations have been suggested for this trend, which can be categorized into natural variability, external variability and observational errors^6^,^7^,^8^. Natural variability includes the El Niño–Southern Oscillation (ENSO) and its decadal variability^9^,^10^, the Pacific Decadal Oscillation (PDO)^3^,^11^, the Interdecadal Pacific Oscillation (IPO)^12^ and trans-basin transportation of mass and energy^9^,^13^,^14^,^15^. Atlantic-warming-induced easterly wind anomalies over the Indo-western Pacific and westerly wind anomalies over eastern Pacific^16^, thereby produce Indo-western Pacific warming and then enhance Walker circulation together by strengthening Pacific trade winds^17^,^18^,^19^, ocean-atmosphere dynamical interactions^20^, and ocean heat storage exchange over Indo-Pacific-Atlantic-Southern oceans^9^,^13^,^14^,^15^. External variability, mainly includes weakening solar activity^21^,^22^,^23^, increasing stratospheric aerosols^24^,^25^,^26^,^27^,^28^,^29^, decreasing stratospheric water vapor concentrations^30^, minor volcanic eruptions^31^ and diminishing sea ice extent^32^. These factors jointly result in a warming slowdown during the period 1998–2013. Furthermore, by analyzing the seasonal mean GMST trends, Cohen, *et al.*^33^ and Trenberth, *et al.*^3^ identified the seasonally asymmetric nature of the temperature trend with evident cooling in winter, which was suggested to be associated with sea surface temperature. However, after adjusting for sea surface temperature anomalies over the equatorial eastern Pacific in a coupled climate model, the GMST trend was reproduced, whereas the winter trend over Eurasia was not^10^.

Although the warming hiatus expressed by GMST has been attributed to the ocean to some extent, the regional components of the warming hiatus and their underlying mechanisms are not well constrained, especially over land. Recently, extreme cold events in winter occurred over the midwestern and southeastern United States (US) and Europe, with strong and cold winds. There are two current views to explain these events. One is that the Arctic warms and the polar vortex weakens as a result of the reduction in sea ice extent, allowing a high volume of cold air to rush into the mid-latitudes as a wave, thereby maintaining the mild temperature in the Arctic, known as “warm Arctic-cold continents”^32^,^34^,^35^,^36^,^37^. The other view is that the increase in summer Eurasian snow cover and the warming Arctic together induce a negative trend in the Arctic Oscillation (AO), which increases the frequency of Eurasian blocking and cools the mid-latitudes^37^,^38^,^39^,^40^,^41^,^42^. Because diminishing sea ice has an evident impact on mid-latitudes temperature variability on an annual timescale, whether and to what extent its spatial pattern may influence the surface warming trend in the most recent decade requires examination.

Most of the existing studies were based on global analyses of *T*_*a*_, including those performed by several groups, such as the National Oceanic and Atmospheric Administration’s (NOAA) National Climatic Data Center (NCDC) with the Global Historical Climatology Network (GHCN)^43^,^44^,^45^, the Goddard Institute for Space Studies (GISS)^46^, and a joint effort between the Met Office Hadley Center and the University of East Anglia Climate Research Unit with Temperature, version 4 (CRUTEM4)^47^,^48^. All of the global temperature analyses for climate detection and attribution over land performed by the aforementioned groups relied heavily on *T*_*2*_^49^,^50^. However, existing studies have reported that observation time and temperature definition do bias daily mean temperature^51^,^52^,^53^,^54^,^55^,^56^,^57^,^58^. For examples, *T*_*a*_ is recorded from midnight to midnight as a day at first order National Weather Service stations, but the observation is usually taken at midmorning or late afternoon at cooperative stations for convenience. The different ‘day’ defined by the observation time leads to varying daily maximum and minimum temperature for a day, which would bias daily mean temperature^54^,^59^,^60^. Despite of a relative small bias for a majority of days, bias in daily mean temperature can be large and of either sign as a large difference in day-to-day temperature^61^. Another case, if close to summer or winter solstices, biases from the two sun-time changes could be included in the records when the observation time is not at midnight, which is verified by Vose, *et al.*^58^. Recent researches have noted that the trend of *T*_*2*_ has notable biases of 25% at a grid scale size of 5° × 5° 2, and the trend bias for the 1973–1997 period can partially explain the enhanced warming rates over the northern high latitudes and the “warming hole” over the central US^62^. Moreover, several studies^7^,^63^,^64^,^65^ have pointed out the underestimated effect of bias from data coverage on the recent warming trend. Therefore, the incomplete spatial sampling of data has a strong impact on the global or regional warming rate. However, whether the temporal sampling bias has an evident impact on the recent warming slowdown and its spatiotemporal pattern still remains unclear.

Daily maximum (*T*_*max*_) and minimum temperature (*T*_*min*_) have been operationally observed at weather stations globally since the middle of the 19^th^ century^66^. Their average (*T*_*2*_ = (*T*_*max*_ + *T*_*min*_)/2) has been taken as a standard definition of *T*_*a*_^1^ and has been the backbone of current global analyses of *T*_*a*_ over land^45^,^47^,^49^. Usually, *T*_*2*_ is applied from 0:00 to 0:00 O’clock daily. Hourly temperature data have increasingly become available since the 1990s as the observing infrastructure has been automated^1^. Mean temperature can also be calculated from 24 hourly observations at local time 
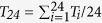
, which has been regarded as the true mean temperature^2^,^49^. This would describe the underlying physical processes better but has rarely been used and evaluated in climate analyses.

Under clear sky conditions, *T*_*a*_ usually reaches *T*_*min*_ in the early morning because of long-wave radiation cooling and reaches *T*_*max*_ in the early afternoon because of solar short-wave radiation heating. However, because the significant diurnal cycle of temperature is easily affected by land-atmosphere states^67^, such as notable variations in incoming solar radiation, indirect/direct aerosol effects, precipitable water vapor^68^, soil moisture, cloud conditions^69^, large-scale circulation modes^70^ and vegetation cover, it is not linear or symmetrical^2^. Therefore, *T*_*2*_ may introduce bias in estimating the true monthly mean temperature. What is the magnitude of this bias and its effect on the warming hiatus for the 1998–2013 period? Does this bias and its effect on temperature trend vary by season and geographic location?

To answer the above questions, we conducted a comprehensive quantitative assessment of the temperature trend difference between *T*_*2*_ and *T*_*24*_ and its spatiotemporal features, to examine its effect on depicting the recent warming hiatus. *T*_*24*_was averaged from the continuous hourly *T*_*a*_ observations collected by the NCDC Integrated Surface Database (ISD-H)^71^, available at approximately 3400 globally distributed weather stations since 1998. Note that warm season is defined from May to October in Northern Hemisphere and from November to April in Southern Hemisphere, as opposite for cold season.

## Results

### Warming Hiatus Contrast over Ocean and Land

**Figure 1:**
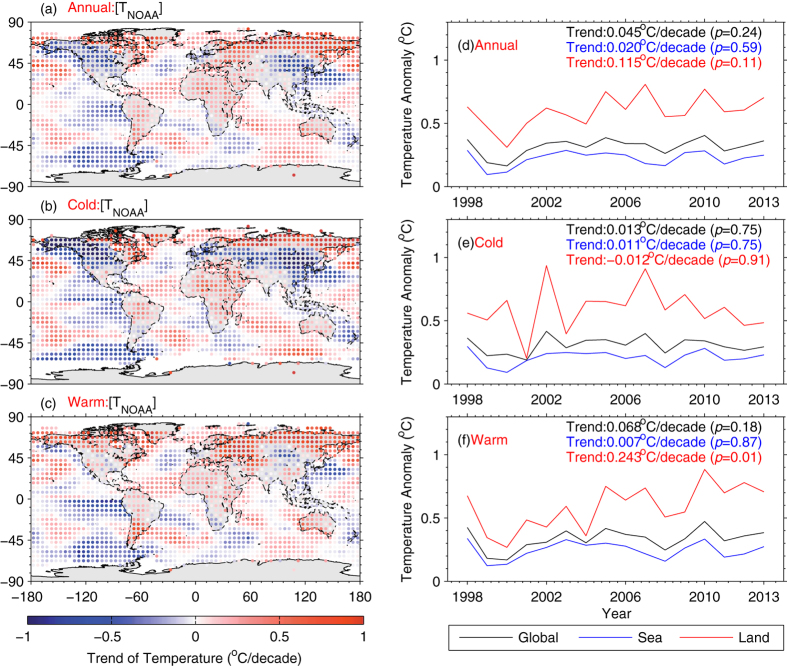
The (**a**) annual, (**b**) cold, and (**c**) warm seasonal temperature trends (units: °C/decade) for 1998–2013 are shown from the Merged Land-Ocean Surface Temperature Analysis (version 3.5.3) of the National Oceanic and Atmospheric Administration (NOAA-MLOST). Changes in the temperature anomalies at (**d**) annual, (**e**) cold and (**f**) warm seasonal timescales are shown over the globe, land and sea. The trends and statistical significance level (*p*) according to the *t*-test method are listed in each panel. This dataset is comprised of land surface temperature from the Global Historical Climate Network (GHCN) and sea surface temperatures from the extended reconstructed sea surface temperature (ERSST) analysis version 3. It manifests a cooling of the eastern and western Pacific along the coast and some regions of the Atlantic Ocean but also a much cooler temperature change in cold seasons over North America and eastern and central Eurasia. Accordingly, the global warming hiatus (0.045 °C/decade) mainly results from the ocean warming slowdown (0.02 °C/decade) and the land cooling in cold seasons (−0.012 °C/decade). In addition, there is a larger divergence in seasonal trends over land than over ocean. This figure was produced by MATLAB version 7.13 (http://www.mathworks.com/products).

Here, we comprehensively described the regionality and seasonality of recent warming hiatus, including contrast of ocean and land surface warming rates. Figure 1 shows the temperature trend for 1998–2013 over ocean and land based on data from the Merged Land-Ocean Surface Temperature Analysis (version 3.5.3) of the National Oceanic and Atmospheric Administration (NOAA-MLOST), consisting of the Global Historical Climate Network (GHCN) over land^45^ and the extended reconstructed sea surface temperature (ERSST) analysis (version 3) over ocean^72^. It manifests the cooling eastern Pacific in the twenty-first century reported by Kosaka and Xie^10^ as an ENSO-like pattern (Fig. 1a–c) but also indicates much lower temperatures in cold seasons over North America and the mid-latitudes of Eurasia (Fig. 1b), resulting in a more evident hiatus during the period of 1998–2013 over land than ocean. Moreover, the western Pacific along the coast^73^ and some regions of the Atlantic Ocean exhibit cooling in both seasons (Fig. 1a–c). Accordingly, the global warming hiatus for 1998–2013 (0.045 °C/decade, Fig. 1d) mainly results from the ocean warming slowdown over the whole year (approximately 0.01 °C/decade, Fig. 1d,e,f) and the land cooling in cold seasons (−0.012 °C/decade, Fig. 1e).

**Figure 2:**
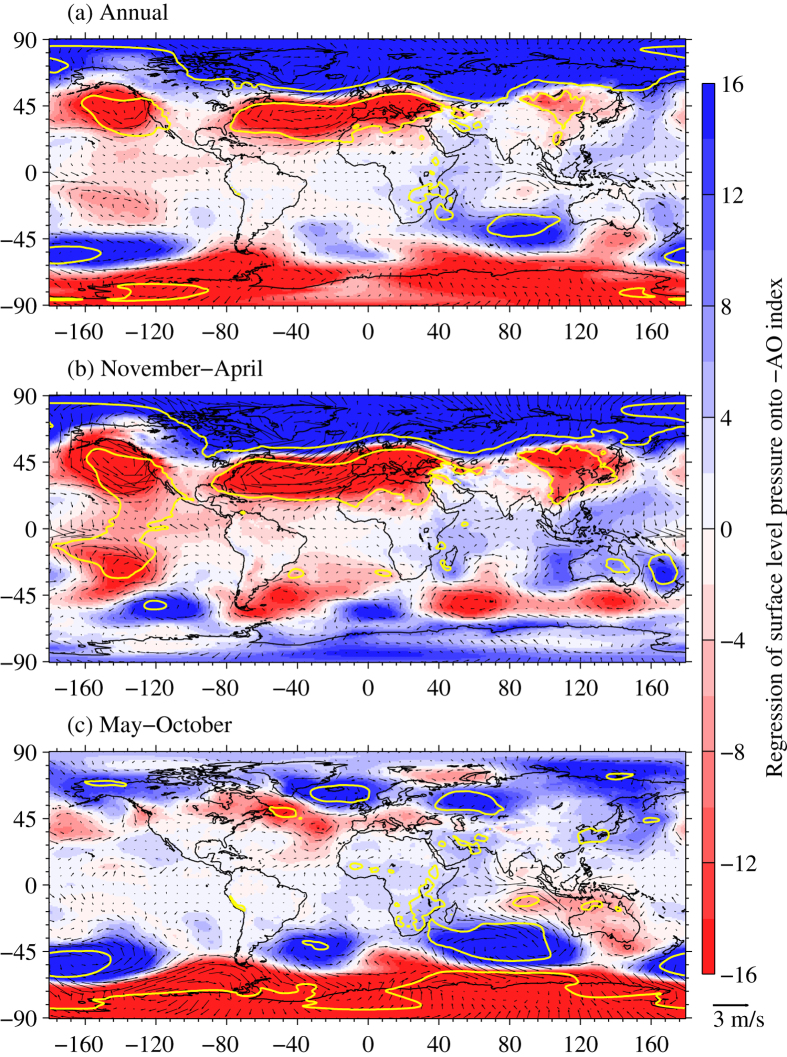
(**a**) Annual-, (**b**) boreal cold (from November to April) and (**c**) warm (from March to October) mean surface pressure anomalies (Unit: m, contours) and 10 m wind anomalies (Unit: m/s, vectors scaling at bottom-right) in ERA-Interim regressed onto the standardized monthly inverted Arctic Oscillation index (-AO) during the period of 1998–2013. Yellow contours indicate a significant level of 0.1. This figure was produced by MATLAB version 7.13 (http://www.mathworks.com/products).

There is a larger spatial pattern of temperature trends over land than over ocean, including evident cooling over midwestern North America and the mid-latitudes of Eurasia, but enhanced warming north of 50°N even in cold seasons, i.e., the “warm Arctic-cold continents” pattern (Fig. 1a–c). There are two dynamic processes accounting for their regional contrast: (1) the intensified trade winds over the equatorial Pacific pushed warm water into the western Pacific (an ENSO-like pattern modulated by the PDO at a decadal timescale), forming quasi-stationary Rossby waves in the upper troposphere, which in turn influence the Arctic and force the North Atlantic Oscillation (NAO) into its negative phase^3^. This favors the formation of cold weather systems in Europe and the southern US, which helps to explain the cooling; (2) the negative-phase AO significantly exhibiting higher than normal Arctic pressure and lower than normal subtropical pressure centered in mid-latitudes (Fig. 2a,b), favors the southward transport of cold air from the Arctic to the mid-latitudes of Eurasia. Cohen, *et al.*^40^ and Wallace, *et al.*^74^ have linked the recent winter cooling to the surface circulation. Mori, *et al.*^37^ regressed near-surface wind fields and surface level pressure onto the leading modes of surface *T*_*a*_ that closely correlate with AO index and sea ice decline, and suggested that negative AO phase increases the probability of severely cold winter in mid-latitudes of Eurasia continent, independent of impact of sea ice decline. Huang, *et al.*^75^ further revealed the decadal effect of oscillations in winter cooling over Northern Hemisphere land and Guan, *et al.*^76^ successfully separated the radiative and dynamical changes in *T*_*a*_, including NAO, PDO and Atlantic Multidecadal Oscillation (AMO).

We further show surface wind anomalies associated with the negative-phase AO during the period of 1998–2013 in Fig. 2. First, northerly wind anomalies from Laptev Sea spin southward into mid-latitudes and then westward into North Europe (Fig. 2b). This process carries cold air in Arctic into mid-latitudes to cause surface cooling in winter of the most recent decade (Fig. 1b). Second, the negative-phase AO enhances propagation of northerly wind anomalies onto northeast Pacific and northwest Atlantic oceans (Fig. 2b,c), providing cold source for these regions and Alaska in both seasons (Fig. 1b,c). Despite an insignificant trend of AO during the period 1998–2013, the shift into negative phase of AO (−0.077, −0.072 and −0.035 for annual, cold and warm seasons, respectively) favors the advection of cold air to the mid-latitudes of Eurasia, leading to recent winter cooling (Fig. 1), compared to the result of Mori, *et al.*^37^. Third, stronger than normal westerlies carry warm and humid air over open ocean into South Europe and North Africa (Fig. 2b) and then warm these regions during cold seasons (Fig. 1b). Finally, the 10 m wind anomalies originating from Barents-Kara sea (cold air) is directed southward to northern Atlantic ocean and arcs north into the Canadian Arctic (mild oceanic air) (Fig. 2), partly leading to the warming around the Canadian Arctic (Fig. 1).

**Figure 3:**
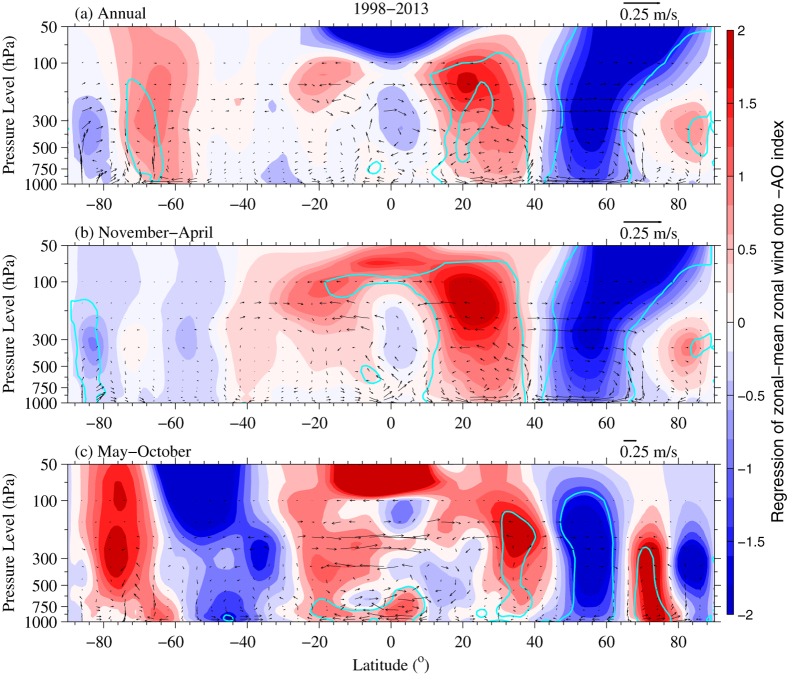
Zonal-mean zonal wind anomalies (Unit: m/s) regressed onto the standardized monthly Arctic Oscillation index (AO), expressed as filled contours at (**a**) annual, (**b**) boreal cold (from November to April) and (**c**) warm (from March to October) seasonal timescales during the period of 1998–2013. The red filled contours are westerly wind anomalies whereas blue ones are easterly wind anomalies. Cyan contours indicate a significant level of 0.1. Composite vertical-meridional circulation anomalies regressed onto the AO index are shown in vectors consisting of vertical velocity and meridional flow anomalies (Unit: m/s, scaling at top-right). The data used here are from pressure fields of the ERA-Interim at 1° × 1° global grids. This figure was produced by MATLAB version 7.13 (http://www.mathworks.com/products).

In order to clarify the dynamical process that the AO influences surface cooling in most recent decade in mid-latitudes, three-dimensional structure of wind field associated with AO is supplemented. Figure 3 shows the zonal-mean zonal wind and vertical-meridional circulation anomalies regressed onto the inverted AO index from 50 to 1000 hPa. Zonal-mean zonal wind anomalies associated with AO exhibits a meridional dipole between approximately 10°N and 70°N, i.e., easterly anomalies in mid-and high- latitudes and westerly anomalies in subtropics (Fig. 3a,b), presenting a equivalent barotropic structure of AO revealed by the original works of Thompson, *et al.*^77^ and Thompson and Wallace^78^. Specifically, extent of the barotropic structure in boreal cold seasons is larger than that in boreal warm seasons (Fig. 3a,b). Westerly anomalies have a maximum at ~200 hPa of ~25°N in boreal cold seasons and at ~35°N in boreal warm seasons, whereas easterly anomalies enhance along the geopotential height so as to reach a maximum at ~50 hPa of ~70°N in boreal cold seasons and at ~300 hPa of ~50°N in warm seasons during the period of 1998–2013.

The zonal-mean vertical meridional circulation anomalies associated with AO exhibit a tripole structure of anomalous Hadley-Ferrel-Polar cells in boreal cold seasons (Fig. 3b). Similar to surface wind anomaly fields, anomalous flow at ~800–1000hPa in the Arctic goes downward into mid-latitudes along the lower branch of anomalous Ferrel cell and propagates westward North Europe (Fig. 3b). A considerate part of cold air is mixed with warm and dry air that is carried by the upper branch of Hadley cell and subsiding at ~35–45°N. A weakened lower branch of Hadley cell makes it possible for cold air directly go southward and eastward into South Asia (Figs 2b and 3b), resulting in surface warming slowdown in South Asia (Fig. 1b). Mild air at ~200–500 hPa over tropics and subtropics is transported by the upper branch of anomalous Ferrel cell into the Arctic and subsides north of ~75°N (Fig. 3b), which amplifies the Arctic warming in the last decade (Fig. 1b). However, because Hadley cell moves northward and Ferrel cell shrinks, it leads to poleward propagation of low-level meridional flow north of ~65°N (Fig. 3c), and so enhanced Arctic warming also occurs in warm seasons (Fig. 1c). Anomalous meridional flow at ~100–500 hPa originates from the Arctic and subsides in ~60°N latitude (Fig. 3c), resulting in slight cooling in central Asia (Fig. 1c). Therefore, the spatial pattern is closely associated with climate oscillation indices, i.e., the interannual variability of the NAO, the AO^76^,^79^, intensified trade winds^19^, and an ENSO-like mode^3^,^10^,^11^,^80^. In addition, atmospheric aerosols, especially over the Arctic and China, have altered the recent warming rate^24^,^25^,^26^,^27^,^28^,^29^,^81^. Land use changes in the form of deforestation, agriculture and urbanization have an important role in local cooling^82^,^83^,^84^,^85^,^86^.

In all, a larger divergence in seasonal trends is illustrated over land than over ocean. Equivalent temperature trends in cold and warm seasons occur over the surface ocean (−0.011 °C/decade and −0.007 °C/decade, respectively), whereas a low-temperature trend of −0.012 °C/decade occurs over land during cold seasons, which was even lower since 2006. In addition, a high value of 0.243 °C/decade is present over land during warm seasons (Fig. 1e,f), resulting from anthropogenic influence and natural interannual variability^87^,^88^. These results reveal the seasonal and regional (or sea-land) aspects of the hiatus.

### Warming Hiatus Associated with the Definition of Temperature

**Figure 4:**
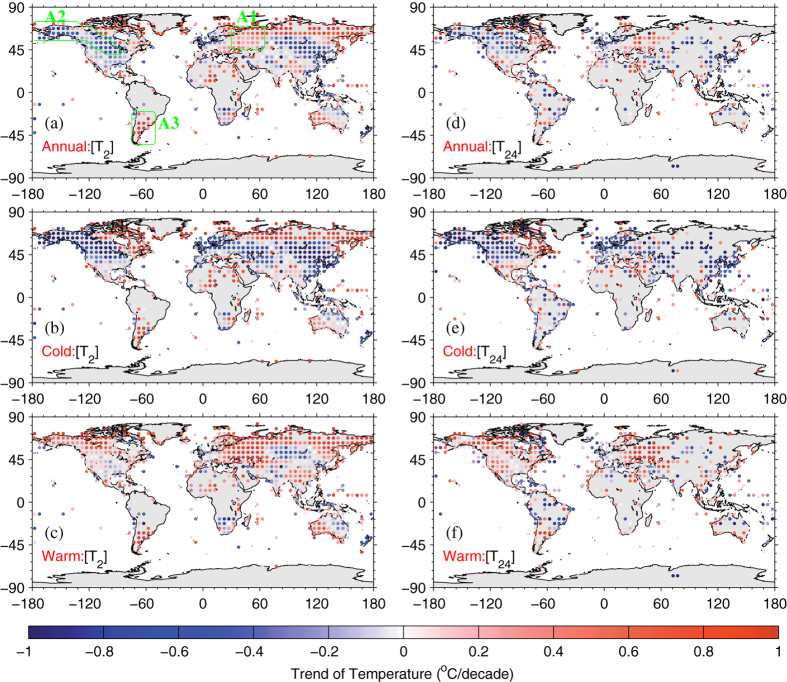
The (**a**,**d**) annual, (**b**,**e**) cold, and (**c**,**f**) warm seasonal temperature trends (unit: °C/decade) from the Global Historical Climatology Network-Daily version 3.2 (GHCN-D, [*T*_*2*_]) and the Integrated Surface Database-Hourly (ISD-H, [*T*_*24*_]) are shown for 1998–2013. The GHCN-D is an integrated database of daily climate summaries from land surface stations across the globe, which provides available *T*_*max*_ and *T*_*min*_ at approximately 10,400 stations from 1998 to 2013. The ISD-H consists of global hourly and synoptic observations available at approximately 3400 stations from over 100 original data sources. Regions A1, A2 and A3 (inside the green regions shown in the top left subfigure) are selected in this study. This figure was produced by MATLAB version 7.13 (http://www.mathworks.com/products).

**Figure 5:**
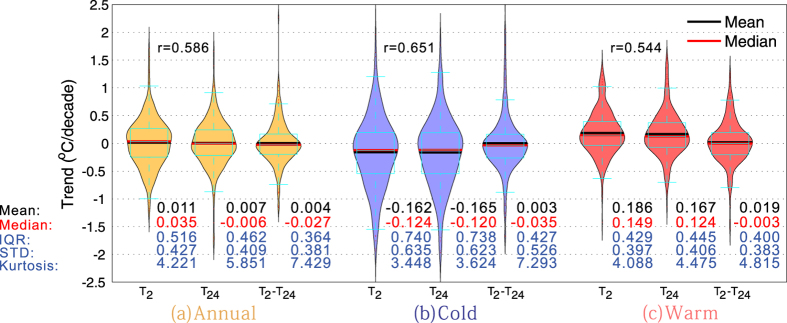
Distributions of the trends in *T*_*2*_ and *T*_*24*_ and their difference (expressed as *T*_*2*_-*T*_*24*_) over (**a**) annual, (**b**) cold and (**c**) warm seasons in a “violin” diagram, showing the mean (black horizontal line), median (red horizontal line), interquartile range (box with 1.5 times whiskers), and the probability distribution (brown, light blue and pink violins). The trends in *T*_*2*_ and *T*_*24*_ are to promote the statistics analysis based on a same spatiotemporal cover. The numbers inside the figure denote the mean (in black), median (in red), standard deviation (STD), and kurtosis (in light blue) of the *T*_*2*_ and *T*_*24*_ trends and their differences for the 1998–2013 period. The spatial correlations (*r*) are embedded inside the top of the figure. The figure was produced in MATLAB v7.13 (http://www.mathworks.com/products).

To determine whether different temperature definitions have a considerable impact on the warming hiatus in terms of its spatial pattern and seasonal variance, Fig. 4 shows a map of the trends of *T*_*2*_ and *T*_*24*_ for 1998–2013. They show similar overall spatial patterns (left v.s. right columns of Fig. 4), but the spatial correlations between the trends of *T*_*2*_ and *T*_*24*_ at a grid of 5° × 5° are low at 0.59, 0.65 and 0.54 over annual mean, cold and warm season means, respectively (Fig. 5). However, the spatial pattern of temperature trends in cold seasons (interquartile ranges (IQRs) of 0.740 °C/decade for both *T*_*2*_ and *T*_*24*_) is more evident than that in warm seasons (IQRs of 0.43 °C/decade and 0.45 °C/decade for *T*_*2*_ and *T*_*24*_, respectively) (Figs 4b,c,e,f and 5b,c). However, the peaks of the trend distributions for both *T*_*2*_ and *T*_*24*_ are above the median (or mean) in cold seasons but below the median in warm seasons, indicating a different pattern for the two seasons (Fig. 5b,c). The overall warming occurs during warm seasons (means of 0.19 °C/decade and 0.17 °C/decade for *T*_*2*_ and *T*_*24*_, respectively), and an evident slowdown occurs during cold seasons (mean of approximately −0.16 °C/decade for *T*_*2*_ and *T*_*24*_) (Figs 4b,c,e,f and 5b,c), also reflected in the specific regions listed in Table 1. Moreover, the temperature trend has a longer tail in cold seasons (bottom whiskers of −1.65 °C/decade and −1.51 °C/decade for *T*_*2*_ and *T*_*24*_, respectively) than in warm seasons (bottom whiskers of −0.61 °C/decade and −0.69 °C/decade for *T*_*2*_ and *T*_*24*_, respectively), revealing an extreme cooling in cold seasons (Fig. 5b,c). Simultaneously, the top whisker of the temperature trend is slightly higher in cold seasons (1.32 °C/decade and 1.28 °C/decade for *T*_*2*_ and *T*_*24*_, respectively) than in warm seasons (1.04 °C/decade and 0.97 °C/decade for *T*_*2*_ and *T*_*24*_, respectively), indicating notable warming over some regions occurring in cold seasons (Fig. 5b,c).

**Figure 6:**
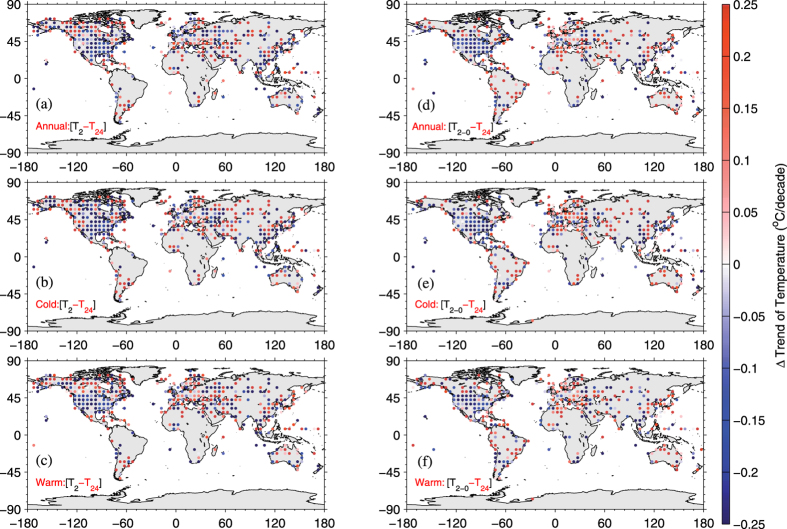
Annual (**a**), cold (**b**) and warm (**c**) season trend differences between *T*_*2*_ and *T*_*24*_ are shown. *T*_*2*_ is averaged from *T*_*max*_ and *T*_*min*_, whereas *T*_*24*_ is averaged from 24 hourly temperature measurements from midnight to midnight local time. To show the small impact of data homogenization on trend differences between *T*_*2*_ and *T*_*24*_ during the period of 1998–2013, trend differences between *T*_*2–0*_ and *T*_*24*_ are shown in right column. Trend in *T*_*2–0*_ is calculated from homogenized dataset, i.e. the Global Historical Climatology Network-Monthly (*GHCN-M*, version 3.2.2), which has been carried out the temporal-spatial consistency check and frequent-value check etc., but involves only fewer stations than the daily base. Similar patterns in right column are obtained to indicate that the consistency check has little impact on trend differences for the period of 1998–2013. This figure was produced by MATLAB version 7.13 (http://www.mathworks.com/products).

The differences in their trends, where were calculated when both *T*_*2*_ and *T*_*24*_ were available, exhibit notable regionality (Fig. 6). A markedly underestimated *T*_*2*_ trend occurs in America and the eastern European plain (A1), whereas overestimation of the *T*_*2*_ trend occurs over the western North America (including A2), the surrounding areas of Iceland, southern South America (A3), and arid areas in Eurasia, China, Japan, and Australia (Fig. 6 and Table 1). The consistency of spatial patterns between the trend difference in *T*_*2*_ relative to *T*_*24*_ and the warming rate of *T*_*a*_ indicates that they were impacted by the same key parameters.

Besides, the land-atmosphere interactions, impacted by both large-scale atmospheric circulation and changes in local surface conditions, play an important role in the diurnal variance of surface air temperature. Studies on attribution of warming hole in central and south U. S. revealed that a circulation-precipitation coupling and aerosols can suppress Tmax^68^,^89^. A circulation-precipitation replenishes soil moisture so as to increase local evapotranspiration and aerosols indirectly decrease shortwave cloud forcing by modify cloud optical properties, thereby both suppress Tmax, leading to the underestimated trend of *T*_*2*_ in US (Fig. 6). Under global warming, the decrease in soil moisture in arid and semi-arid regions alters the moisture recycling directly via the portioning of available energy into latent and sensible heat fluxes and vegetation growth^90^,^91^. More available energy partitioned into the sensible heat flux could enlarge Tmax and then lead to the overestimated trend of *T*_*2*_ in arid and semi-arid regions (Fig. 6). During the negative phase of NAO/AO, the propagation of Arctic-originated cold air makes Tmax become lower and substantially alters the diurnal cycle of temperature over Canadian Arctic and Europe, leading to the overestimated trend of *T*_*2*_ in these regions. In addition, weather events, such as front activity and rainfall, could change the diurnal cycle of temperature and then bias the trend between *T*_*2*_ and *T*_*24*_.

The difference between the *T*_*2*_ and *T*_*24*_ trends is more obvious in the Southern Hemisphere: −0.018 °C/decade for *T*_*24*_vs. 0.282 °C/decade for *T*_*2*_. This difference occurred in both the warm season (0.003 °C/decade for *T*_*24*_vs. 0.251 °C/decade for *T*_*2*_), and the cold season (−0.082 °C/decade for *T*_*24*_vs. 0.115 °C/decade for *T*_*2*_) (Table 1). Significantly overestimated annual and seasonal trends (annual trend of 0.71 °C/decade) of *T*_*2*_ relative to *T*_*24*_ (annual trend of 0.18 °C/decade) were found over region A3 (Table 1). The data coverage of the Southern Hemisphere was limited (Fig. 4). The diurnal cycle of temperature is easily influenced by the ocean currents and clouds due to less land in the Southern Hemisphere, which leads to the large trend difference between *T*_*2*_ and *T*_*24*_.

Furthermore, the spatial pattern of the *T*_*2*_ trend is slightly larger than that of the *T*_*24*_ trend, especially at an annual timescale (kurtosis values of 4.22 and 5.85, respectively) (Fig. 5a). The trend difference in *T*_*2*_ and *T*_*24*_ has a standard deviation of 0.38 °C/decade, which is similar to the individual standard deviations (0.43 °C for *T*_*2*_ and 0.41 °C for *T*_*24*_) at an annual timescale. Moreover, the temperature trend in the USA increases from 0.13 °C/decade for *T*_*2*_ to 0.28 °C/decade for *T*_*24*_ but decreases from 0.17 °C/decade for *T*_*2*_ to 0.07 °C/decade for *T*_*24*_ in China for warm seasons (Table 1). This results in the ratio of the warming slowdown over US to that over China decreasing from ~2 to 1 (Table 1) at an annual timescale.

Seasonal divergence is displayed in the trend differences between *T*_*2*_ and *T*_*24*_. The warming rate is better depicted by *T*_*24*_ (mean of 0.17 °C/decade and median of 0.12 °C/decade) than by *T*_*2*_ (mean of 0.19 °C/decade and median of 0.15 °C/decade) in the warm season in terms of the warming hiatus (Fig. 5b,c and Table 1). The kurtosis of the trend difference is greater in the cold season (7.30) than in the warm season (4.82), showing a smaller trend difference in *T*_*2*_ and *T*_*24*_ for the cold season (Fig. 5b,c). This seasonal divergence is observed regardless of the specific region, including the Northern Hemisphere, Southern Hemisphere, and Europe (Table 1).

For a global average (with incomplete coverage), *T*_*2*_ has an important error of annual trend (0.027 °C/decade) with respect to *T*_*24*_ (0.002 °C/decade) during the period 1998–2013 (Table 1). In warm seasons, *T*_*2*_ overestimates the trend by 0.078 °C/decade (approximately 57%), relative to *T*_*24*_ (Table 1). Therefore, the use of *T*_*2*_ may bias the temperature trend over globe and regions, whereas the use of *T*_*24*_ can objectively depict the warming hiatus, rather than *T*_*2*_.

In summary, previous studies^7^,^63^,^64^,^65^ have pointed out the underestimated effect of bias from data coverage on the recent warming trend. Here, we evaluated the effect of bias of *T*_*2*_ on the trend during the warming hiatus compared to that of *T*_*24*_. The trend differences in *T*_*2*_ and *T*_*24*_ exhibit an evident divergence in terms of both regionality and seasonality, which is largely impacted by ocean and atmospheric circulations, including the NAO, the AO and ENSO-like modes, as well as local land-atmosphere interactions from latent and sensible heat fluxes. The recent warming hiatus may be better understood considering these two aspects and biases. Thus, short-duration and regional climate change studies should use high spatiotemporal temperature datasets, such as ISD-H.

### Conclusions and Discussion

This study reviews the warming hiatus and identifies the key factors determining the spatial pattern of the warming hiatus. The warming hiatus over land is more notable in the US, Canada and the mid-latitudes of Eurasia and is especially evident in winter. This warming hiatus may be closely related to the negative phases of the NAO and AO and significant propagation of cold air into mid-latitudes by the northerly wind anomaly originating from the Arctic. The three dimensional structure of circulation associated with AO is first investigated here to clarify the dynamic processes leading to recent winter cooling in mid-latitude of Northern Hemisphere land.

Compared with *T*_*24*_, the use of *T*_*2*_ has a significant error of 0.004 ± 0.38 °C/decade in describing spatial variance of the warming rate for 1998–2013. This indicates that *T*_*2*_ can depict the global average of warming rates but is significantly biased at a regional scale. The bias of the trend in *T*_*2*_ relative to *T*_*24*_ displays spatiotemporal divergence, i.e., significant underestimation over the US but overestimation over the midwest North America, the Arctic, Northern Africa, boreal Eurasia, China and Japan; the *T*_*2*_ trend shows a markedly higher overestimation in warm seasons (by ~57%) than in cold seasons (by ~3%) both regionally and globally; larger spatial incoherence is observed in terms of their difference in warm seasons (kurtosis of 7.3) than in cold seasons (kurtosis of 4.8). This leads to the positive bias in the recent warming hiatus, whereas previous studies^7^,^63^,^64^,^65^ have noted the effect of bias from data coverage leading to underestimation of the recent warming trend. The recent warming hiatus may be better understood by considering this spatiotemporal divergence and the biases.

A more notable warming slowdown occurs over land compared to that over ocean, especially over the midwest North America and Mideast Eurasia in cold seasons, which cannot be characterized by the average trend of the Coupled Model Intercomparison Project Phase 5 (CMIP5)^12^. However, because modelers do not use the true monthly mean temperature to evaluate their models, it is unclear whether climate models can reconstruct the recent warming hiatus itself.

In a word, the above analysis reveals the use of *T*_*2*_ not only leads to a positive bias in the recent warming hiatus, but enlarges the spatial divergence of temperature trend. This has an important implication for the assessment of reanalyzed and modeled temperatures whose outputs are at sub-hourly or hourly timescales. Therefore, we encourage using hourly temperature measurements for the detection and attribution of short-duration and regional climate change based on available historical data, i.e., the ISD-H dataset.

### Data and Methods

The global temperature trend for 1998–2013 was investigated via the Merged Land-Ocean Surface Temperature Analysis (version 3.5.3) of the National Oceanic and Atmospheric Administration (NOAA-MLOST), consisting of the Global Historical Climate Network (GHCN, version 3.2.2) over land^45^ and the extended reconstructed sea surface temperature (ERSST) analysis (version 3) over ocean^72^. Mean temperature data were provided at a grid scale size of 5° × 5°. Note that Antarctic continent is excluded in all the analysis of this study.

To investigate the impact of temperature bias on the recent warming hiatus, the Global Historical Climatology Network Daily (GHCN-D) database, which provides *T*_*max*_ and *T*_*min*_ from approximately 10,400 stations from 1998 to 2013, was used to calculate *T*_*2*_ from midnight to midnight local time^92^, and the NCDC Integrated Surface Database Hourly (ISD-H), which consists of global hourly and synoptic observations available at approximately 3400 stations from over 100 original data sources, was used to calculate *T*_*24*_^71^. *T*_*24*_ was calculated from the integral of the continuous temperature measurements, i.e., 24 hourly temperature measurements from midnight to midnight local time. Calculation of *T*_*2*_ and *T*_*24*_ over the same 24 hr period can eliminate the effect of differently defined ‘day’ (see Section in Introduction). Their temperature anomalies relative to the 2001–2010 period were calculated. To reduce spatially and temporally non-random coverage bias^48^, the absolute values at all available stations were converted to anomalies relative to the 2000–2010 average, gridded into 1° × 1° grids, and then re-gridded into 5° × 5° grids. Prior to gridding the data, it requests the data length of stations to has no less than 15 days for each month, 90 days for cold and warm seasons and 16 years for study period. In addition, these *T*_*2*_ and *T*_*24*_ values were compared only at grids where station data were available without any temporal and spatial interpolation according to NOAA-MLOST.

In order to elaborate the impact of data homogenization on trend differences between *T*_*2*_ and *T*_*24*_ during the period of 1998–2013, the Global Historical Climatology Network-Monthly (*GHCN-M*, version 3.2.2) is used to calculate monthly temperature as *T*_*2–0*_^45^. The *GHCN-M* has been promoted the temporal-spatial consistency check and frequent-value check etc. But the *GHCN-M* involves only fewer stations than *GHCN-D* does^45^,^93^. Similar results obtained from *GHCN-M* (Fig. 6a,b,c) to those from *GHCN-D* (Fig. 6d,e,f) indicate that the consistency check has little impact on trend differences for the period of 1998–2013.

In order to reveal the dynamic processes that the AO influences the recent temperature change in mid-latitudes from 1998 to 2013, surface fields including surface pressure and 10 m wind vector, and three dimensional wind fields in the ERA-Interim at 1° × 1° global grids^94^ were used to regress onto the standardized monthly AO index. The AO index is defined as the first leading principal component of monthly mean sea-level pressure (SLP) variances north of 20°N. At the negative phase, the AO index is characterized by higher than normal SLP anomalies around the Arctic and lower than normal SLP anomalies centered at the subtropical and mid-latitudes^95^.

Previous studies^7^,^63^,^64^,^65^ have pointed out that incomplete spatial sampling may bias the recent warming trend over the past decade. In order to avoid impact of the different spatiotemporal covers of *T*_*2*_ and *T*_*24*_, the statistics analysis was carried out for the same spatiotemporal cover. The usual statistical terms, including mean, median and standard deviation (STD), were adopted to depict the trend differences. The interquartile range (IQR) is a measure of statistical dispersion and is equal to the difference between the upper and lower quartiles. Kurtosis is a measure of whether the data are peaked or flat relative to a normal distribution. Because the kurtosis of any normal distribution is 3, distributions with kurtosis less than 3 are platykurtic and those with kurtosis greater than 3 are leptokurtic. The spatial correlation (*r*) was determined as the Pearson correlation of the two arrays, representing the changes over the 5° × 5° global grids.

## Additional Information

**How to cite this article**: Zhou, C. and Wang, K. Spatiotemporal Divergence of the Warming Hiatus over Land Based on Different Definitions of Mean Temperature. *Sci. Rep.* **6**, 31789; doi: 10.1038/srep31789 (2016).

## Figures and Tables

**Table 1:** Comparison of the temperature trends (units: °C/decade) between *T*_*2*_ and *T*_*24*_ for 1998–2013 over nine regions.

Regions	Annual		Cold		Warm	
	[*T*_*2*_]	[*T*_*24*_]	[*T*_*2*_]	[*T*_*24*_]	[*T*_*2*_]	[*T*_*24*_]
Global land (Full)	0.115	—	−0.012	—	0.243^**^	—
Global land	0.027	0.002	−0.166^*^	−0.162^*^	0.215^**^	0.137
Northern Hemisphere	−0.012	0.001	−0.221^**^	−0.185^*^	0.206^**^	0.172^*^
Southern Hemisphere	0.282^**^	−0.018	0.115	−0.082	0.251^**^	0.003
Europe	0.125	0.089	−0.345	−0.361	0.306^**^	0.248^*^
US	−0.432^**^	−0.255	−0.684^**^	−0.640^**^	0.126	0.283
China	−0.233	−0.241^*^	−0.587^*^	−0.484^*^	0.174	0.069
A1	0.309	0.448^*^	−0.494	−0.264	1.071^**^	1.032^**^
A2	−0.451^**^	−0.341^**^	−1.048^**^	−0.991^**^	0.218	0.247
A3	0.714^**^	0.183	0.492^*^	−0.105	0.759^**^	0.434^**^

[*T*_*2*_] and [*T*_*24*_] are the trends for mean temperatures computed from daily maximum and minimum temperatures and continuous hourly temperature measurements, respectively. Regions A1, A2 and A3 are illustrated in Fig. 4a. The bold font with ^**^denotes a 0.05 level of significance, and that with ^*^denotes significance at the 0.1 level (2-tailed). The numbers shown in the first row (Global land (full)) were calculated from all the available data over global land shown in Fig. 1. For comparison, the other numbers were calculated when *T*_*2*_ and *T*_*24*_ covered the same period and same station, as shown in Fig. 6.
